# Supplementary material for: CLEM-Reg: an automated point cloud-based registration algorithm for volume correlative light and electron microscopy
Source: Nat Methods. 2025 Sep 10;22(9):1923–34. doi: 10.1038/s41592-025-02794-0 (PMC12446066; doi:10.1038/s41592-025-02794-0)
Supplement: Supplementary file 1 — Supplementary Notes and Supplementary Figs. 1–5. [file 41592_2025_2794_MOESM1_ESM.pdf]

nature methods

Supplementary information https://doi.org/10.1038/s41592-025-02794-0

CLEM-Reg: an automated point cloud-based registration algorithm for volume correlative light and electron microscopy

In the format provided by the authors and unedited

Supplementary Material: *CLEM-Reg: An automated point cloud based registration algorithm for volume correlative light and electron microscopy*

Daniel Krentzel[1,2], Matouš Elphick[2,3,4,5], Marie-Charlotte Domart[2], Christopher J. Peddie[2], Romain F. Laine[6], Cameron Shand[7], Ricardo Henriques[6,8], Lucy M. Collinson[2] and Martin L. Jones[2]

[1]Imaging and Modeling Unit, Institut Pasteur, Université Paris Cité, Paris, France
[2]Electron Microscopy Science Technology Platform, The Francis Crick Institute, London, UK
[3]Cancer Dynamics Laboratory, The Francis Crick Institute, London, UK
[4]Department of Bioengineering, Imperial College London, London, UK
[5]The Institute of Cancer Research, London, UK
[6]UCL-Laboratory for Molecular Cell Biology, University College London, London, UK
[7]Software Engineering & AI Science Technology Platform, The Francis Crick Institute, London, UK
[8]Instituto de Tecnologia Química e Biológica António Xavier, Universidade Nova de Lisboa, Portugal
R.F.L. current address: Abbelight, 191 avenue Aristide Briand, 94230 Cachan, France

Supplementary Notes

Assessing the robustness of CLEM-Reg to segmentation errors. The CLEM-Reg algorithm requires identification of landmark structures in both FM and EM to obtain an alignment between image volumes. While the segmentation algorithms used in the context of the benchmark data perform well, the robustness of the segmentation algorithms cannot be guaranteed. We reasoned that the deep learning-based segmentation approach using MitoNet would be more susceptible to performance degradation on new EM image volumes and therefore tested the robustness of CLEM-Reg to random loss of mitochondria segmentations across the whole EM volume. The normalised root mean squared error (NRMSE) between CLEM-Reg and manual overlays was computed on Mitotracker and Lysotracker/GFP-TGN-46 channels as a proxy for registration performance. We find that the registration performance is constant up to a loss of around 40% of mitochondria (**Extended Data Fig. 1A**). We also assessed the impact of segmentation errors in different areas of the EM volume and found that the registration performance is more sensitive to the loss of peripheral landmarks, as opposed to losing landmarks in the central region of the EM volume (**Extended Data Fig. 1B**), with the intuition that small fluctuations in rotational transformations are magnified in proportion to the radial distance from the rotational centre.

Benchmarking point sampling frequency and binning. Reducing the number of sampled points from the identified landmarks reduces the registration time. Two parameters control point sampling: sampling frequency and binning. The first of these parameters removes every k^{th} point from the point cloud while the second parameter bins the point cloud by a given voxel size (in pixels) such that points within each voxel are averaged to generate exactly one point. The normalised root mean squared error (NRMSE) between CLEM-Reg and manual overlays was computed on Mitotracker and Lysotracker/GFP-TGN46 channels as a proxy for registration performance. We find that reducing the point sampling frequency from 1/16 to 1/256 with a fixed voxel size leads to a 19-fold decrease (from 33.7 min to 1.8 min) in registration time with no change in registration error. (**Extended Data Fig. 2**). This shows that by tuning the number of points used to represent landmarks, drastic reductions in registration time can be obtained without impacting registration accuracy.

Estimating scaling properties of CLEM-Reg. To find the parameters of the power law that govern the relationship between the number of points used for registration and the time required for registration, values were fitted to a function of the form $y = bx^m$. To better estimate parameters, experimental data was log-log transformed and fitted to the log-log-transformed function $log(y) = m\, log(x) + log(b)$ (**Extended Data Fig. 3**). Values for the exponent m ranged between 1.47 and 1.69 which implies that doubling the number of sampled points increases registration time by a factor of $2^{1.47}$ to $2^{1.69}$ (i.e. 2.77 to 3.23) . This highlights that the limiting steps in scaling up CLEM-Reg are the segmentation and warping, which both scale cubically with the size of the

input volumes, i.e. doubling input dimensions leads to an 8-fold increase in running time for both the segmentation and warping (assuming chunked data).

Performance quantification on endosomes. Firstly, target structures (endosomes in EMPIAR-11537) were manually segmented in 3D in the EM volume in TrakEM2 in Fiji. These endosomes were then cropped with a bounding box from the corresponding manual- or CLEM-Reg-warped FM volumes and then segmented using the Otsu thresholding method to generate the correlating FM segmentation. Segmentations were transformed from pixel space to real space by applying appropriate scaling. The intersected volume between fluorescent and EM segmentations was then computed. Centroid distances were obtained by first constructing meshes from segmentations using marching cubes. Then, centroids were determined on meshes derived from fluorescent and EM segmentations and their Euclidean distance calculated. The size of segmented endosomes was estimated by computing characteristic length scales L from the endosome volume V with $L = \sqrt[3]{V}$ (**Extended Data. Fig. 6**).

Performance quantification on manual landmarks. Landmarks placed by an expert (MCD) were transformed using the rigid transform found with CLEM-Reg and the affine transform found with BigWarp and Euclidean distances between corresponding landmarks placed in EM and those placed in the LM after applying each transform were computed (**Extended Data. Fig. 8A-C**). No significant difference ($p>0.05$ with Student's t-test) between distances for one dataset (EMPIAR-10819) and significant differences ($p<0.05$ with Student's t-test) between distances in two datasets (EMPIAR-11537 and EMPIAR-11666) were observed. Note that the mean difference between distances obtained with CLEM-Reg and manual registration were 196.82±91.86 nm in EMPIAR-11537 and 269.17±159.18 nm in EMPIAR-11666 (orange plots). The mean distance between LM landmarks transformed with CLEM-Reg and BigWarp with respect to landmarks placed in the EM thus falls between the XY and Z resolution of the LM images (120 and 350 nm respectively) for the two datasets where distances were significant.

Manual landmark performance quantification as a function of landmark placement error. To assess robustness of these results to landmark placement errors, landmarks were randomly perturbed by addition of Gaussian noise with $\mu=0$ and σ corresponding to random landmark placement errors in pixels. Euclidean distances between randomly perturbed landmarks placed in EM and LM transformed by CLEM-Reg and BigWarp were then computed and statistical significance calculated with Student's t-test. Measurements for each pixel perturbation were repeated 1,000 times (**Extended Data Fig. 8D**).

Generating CLEM-Reg alignment error maps from manual landmarks. Error maps between the LM landmarks registered by CLEM-Reg (red) versus the EM landmarks (orange) were computed. The error heat maps were obtained by computing thin-plate spline (TPS) transformations to interpolate continuously between the landmarks transformed with CLEM-Reg (red) and the EM landmarks (blue). Then, points were evenly sampled on a grid and the mean square displacement at each sampled point based on the TPS transform was computed and displayed as a heat map (**Extended Data Fig. 9A**). Heat maps are shown as average projections across each spatial axis with projected cell boundaries shown in white (**Extended Data Fig. 9B-D**).

Supplementary Figures

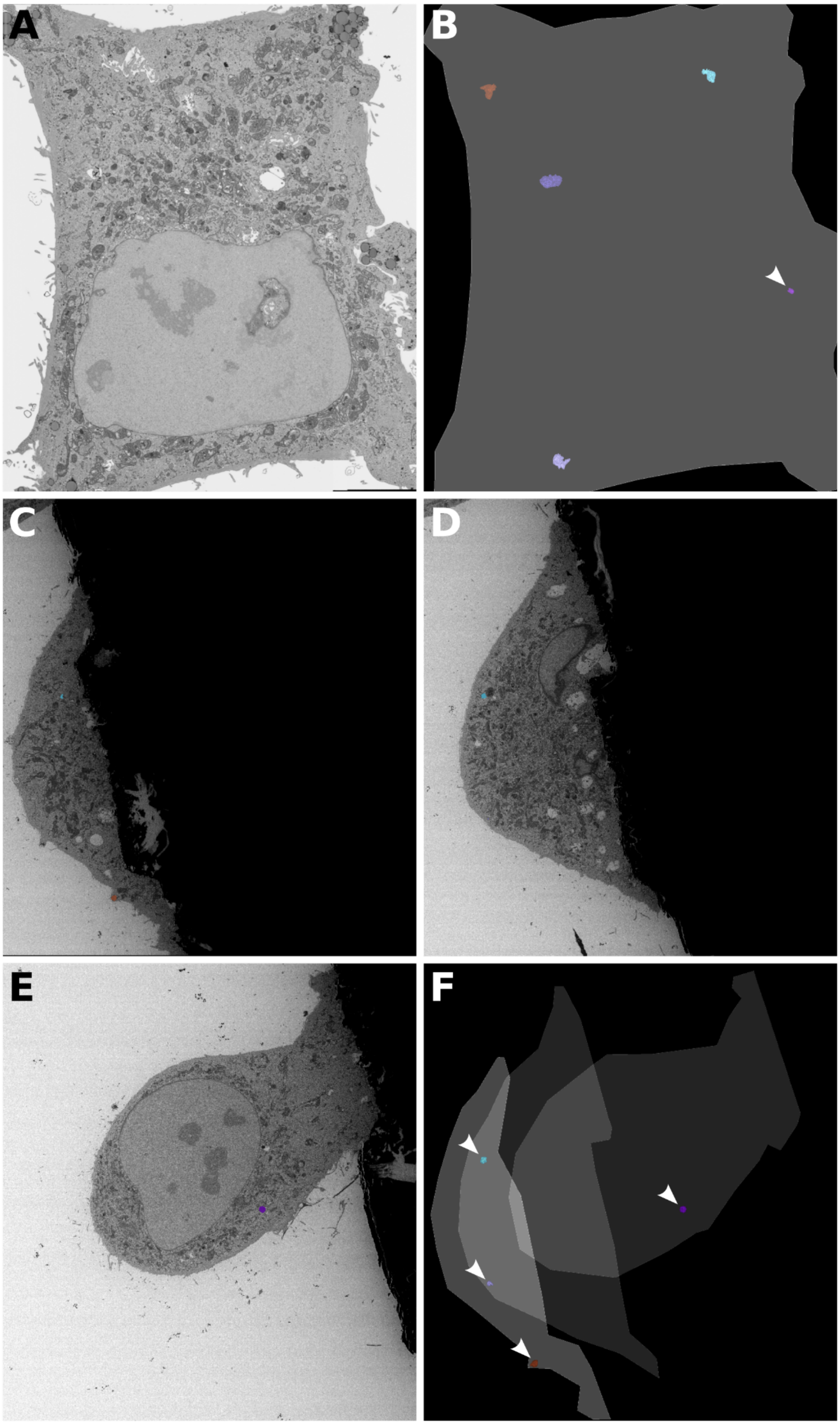

Supplementary Figure 1. Lysosome distribution across EM volumes. (A) Representative FIB-SEM slice from EMPIAR-10819 dataset. (B) 3D rendering of cell outline and five manually segmented lysosomes showing distribution across EM volume. Arrowhead points to a small lysosome. (C-E) SBF-SEM slices from EMPIAR-11666 at different z positions. (F) 3D rendering of cell outlines at different z positions and four manually segmented lysosomes showing distribution across EM volume. Arrowheads point to small lysosomes.

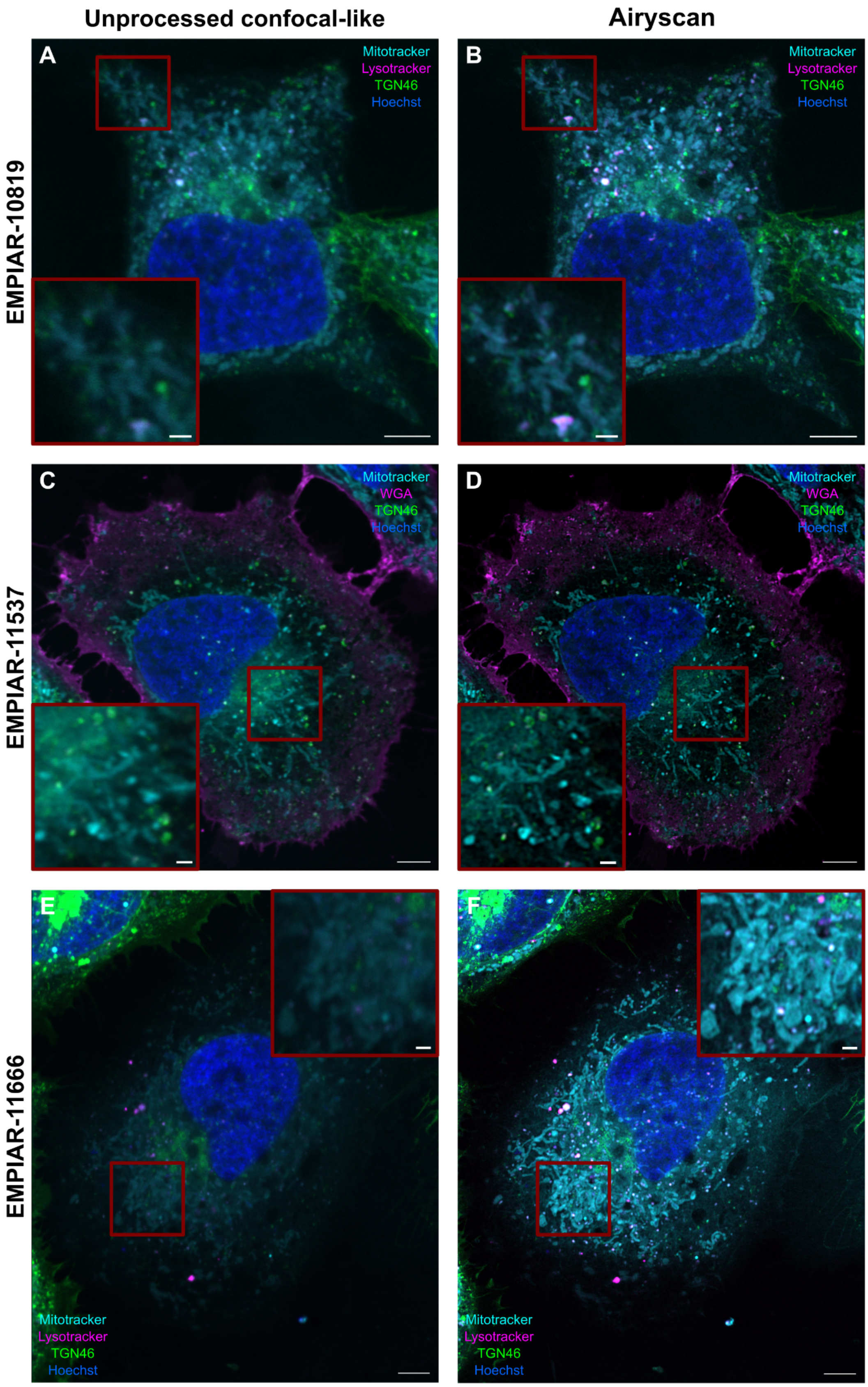

Supplementary Figure 2. Comparison of unprocessed confocal-like and Airyscan data. (A,B) Representative FM slice from EMPIAR-10819 dataset showing unprocessed confocal-like (A) and Airyscan (B) data. (C,D) Representative FM slice from EMPIAR-11537 dataset showing unprocessed confocal-like (C) and Airyscan (D) data. (E,F) Representative FM slice from EMPIAR-11666 dataset showing unprocessed confocal-like (E) and Airyscan (F) data. Scale bars on images are 5 µm and scale bars in insets correspond to 1 µm.

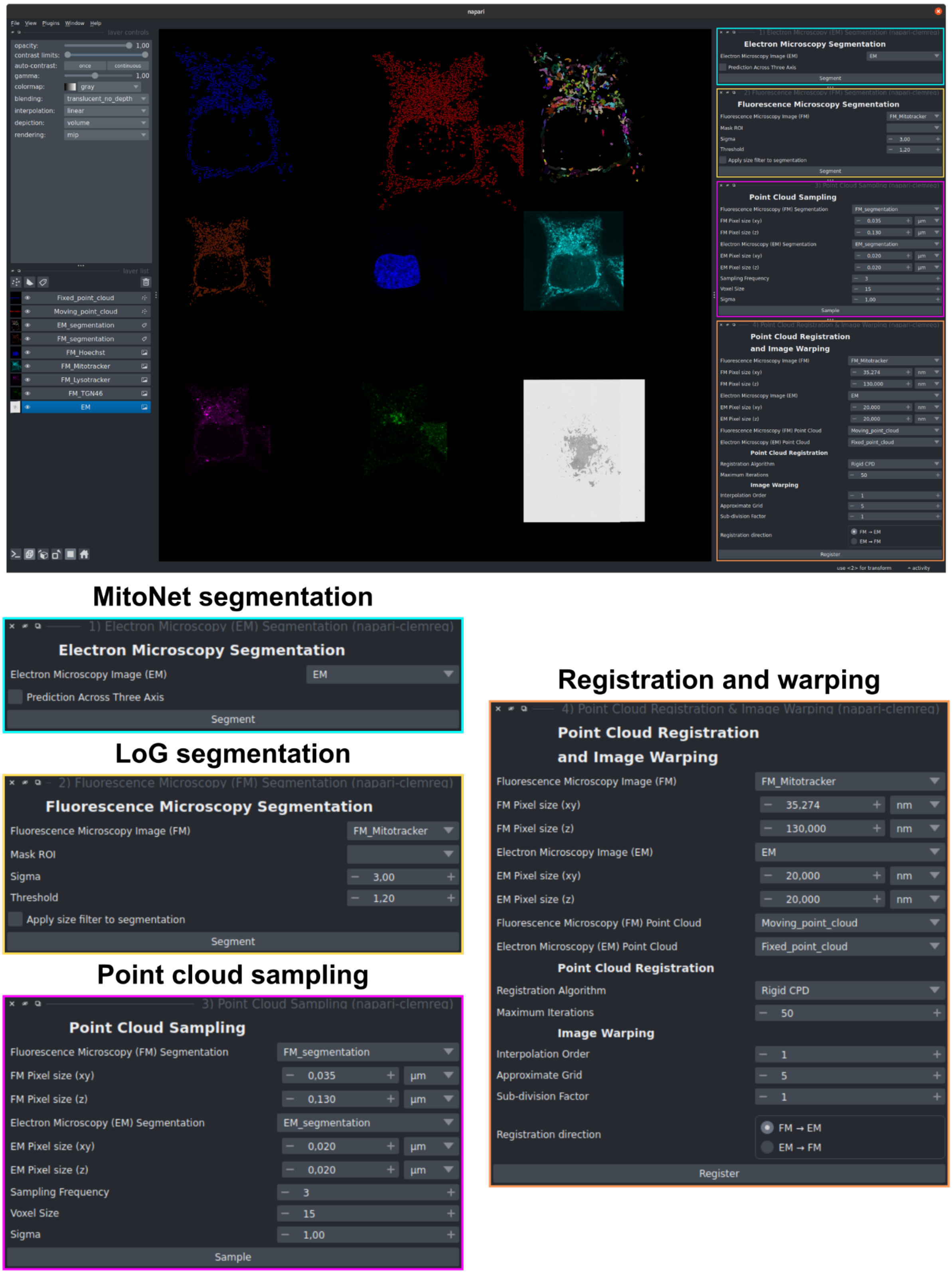

Supplementary Figure 3. Interface of napari-clemreg split registration workflow. Each step of the CLEM-Reg algorithm can be separately executed in the napari-clemreg plugin using the split registration workflow. This enables users to troubleshoot individual steps, inject intermediate results from external sources and interactively tune individual parameters without executing the full pipeline. Specifically, EM and FM segmentation parameters can be tested, point cloud sampling adjusted and registration and warping settings tested.

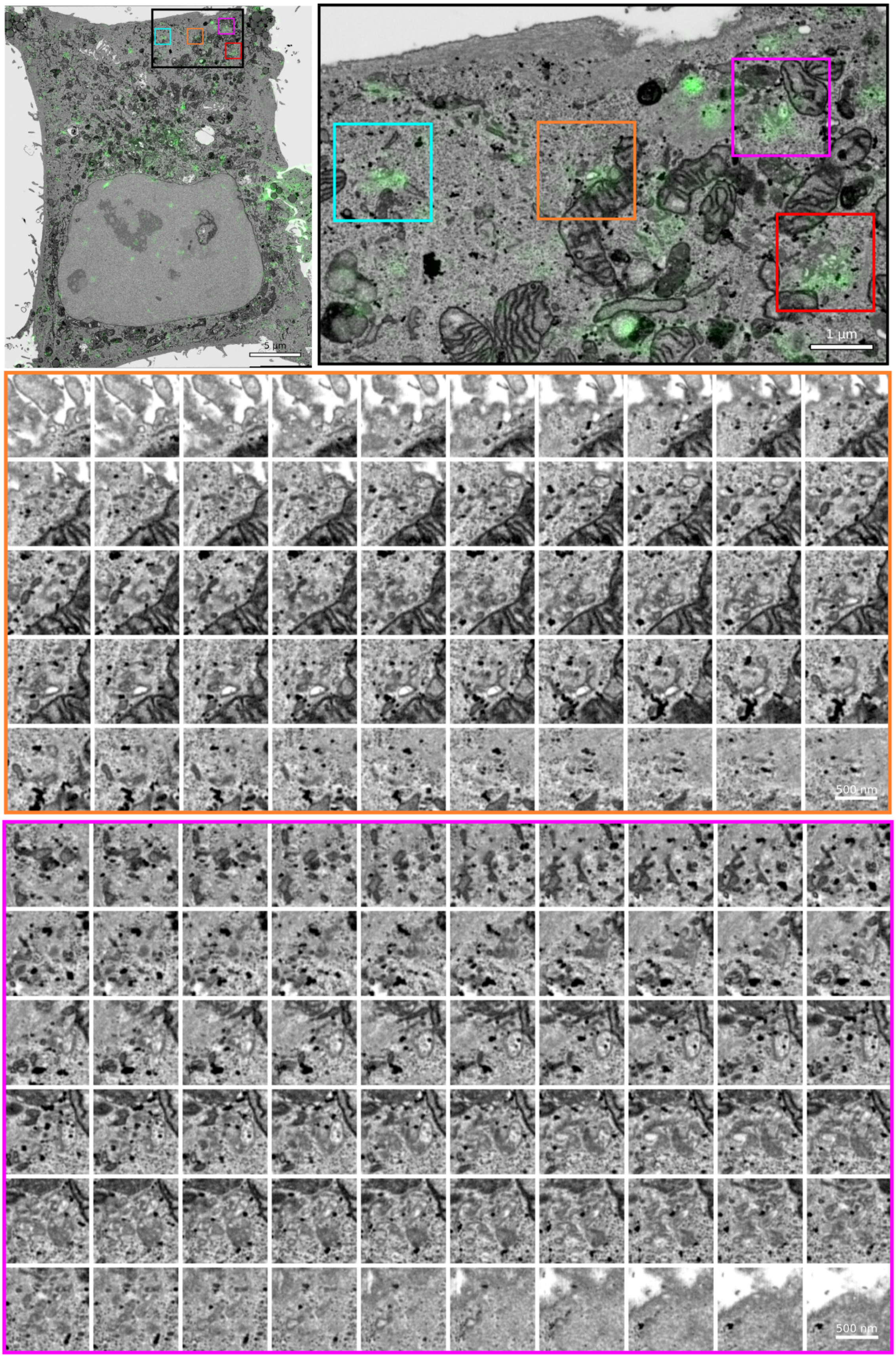

Supplementary Figure 4. Montage of GFP-TGN-46-positive structures in EMPIAR-10819. Full montages of GFP-TGN46-positive structures with 10 nm z spacing on EMPIAR-10819. Movies of GFP-TGN46-positive structures shown in **Supp. Movie 1** (orange) and **Supp. Movie 2** (magenta).

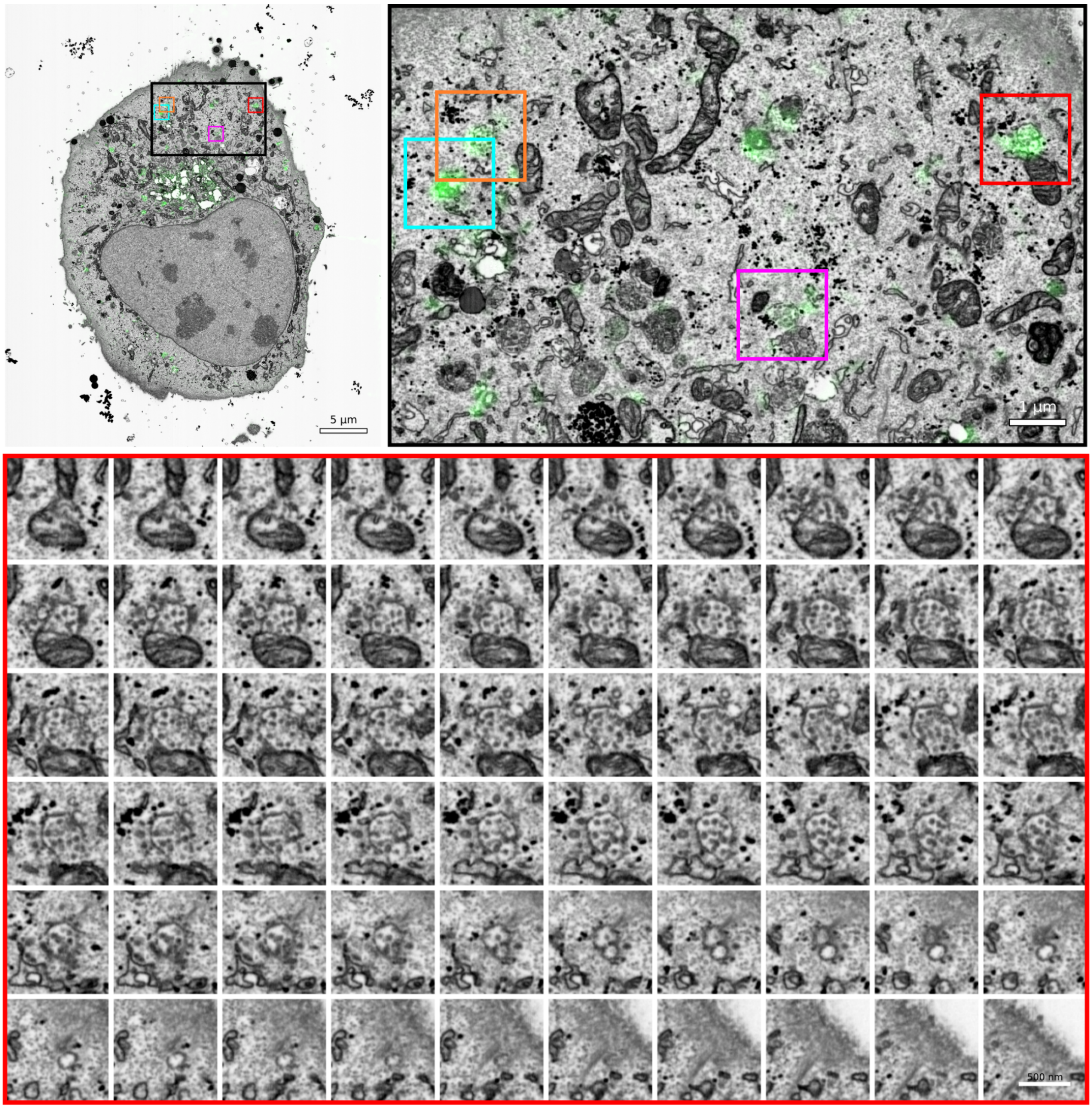

Supplementary Figure 5. Montage of GFP-TGN-46-positive structures in EMPIAR-11537. Full montages of GFP-TGN46-positive structures with 10 nm z spacing on EMPIAR-11537. Movie of GFP-TGN46-positive structure shown in **Supp. Movie 3**.
